# Long term health outcomes in patients with a history of myocardial infarction: A population based cohort study

**DOI:** 10.1371/journal.pone.0180010

**Published:** 2017-07-12

**Authors:** Navdeep Tangri, Thomas W. Ferguson, Reid H. Whitlock, Claudio Rigatto, Davinder S. Jassal, Malek Kass, Olga Toleva, Paul Komenda

**Affiliations:** 1 Department of Medicine, University of Manitoba, Winnipeg, MB, Canada; 2 Seven Oaks Hospital Chronic Disease Innovation Centre, University of Manitoba, Winnipeg, MB, Canada; 3 St. Boniface General Hospital, Department of Cardiac Sciences, Winnipeg, MB, Canada; Thomas Jefferson University, UNITED STATES

## Abstract

**Background:**

Myocardial infarction (MI) is associated with high morbidity and mortality, particularly in the first 12 months post-event. Interventions such as dual antiplatelet therapy can reduce the risk of major adverse cardiovascular events (MACE), but the duration of the high-risk time interval and the optimal prescription time frame for these interventions remains unknown.

**Design, setting, participants, and measurements:**

We performed a retrospective cohort study using data from medical services and hospitalizations in Manitoba, Canada for patients admitted with a MI between April 2006 and March 2010, and followed until Nov 30, 2014. We used survival analysis to determine the cumulative incidence of death, subsequent MI, or stroke, and used Cox proportional hazards models to assess factors associated with these endpoints.

**Results:**

There were 8,493 patients in Manitoba admitted to hospital for a MI during the study period. Of those, 6,749 (79.5%) survived for at least 1 year without a recurrent MI or stroke. In the following year, this population remained at high risk, with 372 (5.5%) of the remaining patients dying in the next twelve months (48.1% cardiovascular deaths), 244 (3.6%) having a recurrent MI, and 74 (1.1%) having a stroke. Older age, male sex, diabetes, prior stroke, prior heart failure, prior unstable angina, and absence of revascularization were associated with worse long-term prognosis.

**Conclusions:**

The risk of MACE remains elevated among post-MI patients after the first year. Interventions to more intensively monitor, evaluate, and treat these patients should be considered beyond the first year following myocardial infarction.

## Introduction

Cardiovascular (CV) disease causes one-third of deaths in Canada, more than any other illness, and is associated with a high economic burden on the health care system [1]. Myocardial infarction (MI), a consequence of CV disease, is associated with significant morbidity and mortality [2]. Despite improvements in acute therapy, and better risk factor control, recurrence rates of major adverse cardiovascular events (MACE), including MI, stroke, and/or death remain high, affecting nearly 1 in 5 patients in the first year post discharge and another 1 in 5 patients over the next 3 years [2–4]. An aging population and increase in obesity rates further threatens to sustain these high rates of MI and CV disease along with their associated adverse events [5].

Secondary prevention efforts are critical in the reduction of MACE in the years following an initial MI [6]. Dual antiplatelet therapy (DAPT) is a mainstay of secondary prevention of MACE up to 1 year post-event [7]. The major complication of DAPT is an increased risk of major bleeding. In clinical practice, DAPT is usually stopped after one year due to a perception of an unfavorable risk to benefit ratio beyond a 12 month timeframe [6]. Recent trials have challenged this assumption and suggested that benefit of DAPT may persist out to 3 years post MI [8]. While recent guideline updates suggest continuation of DAPT may be reasonable in selected patients, they do not otherwise advocate for DAPT use beyond the initial 12 month period [9].

Since most of the therapeutic uncertainty regarding prolonged DAPT hinges on the absolute risk of MACE diminishing after the first post-event year, we sought to address this debate by examining the risk of CV events and mortality, beyond the first post-event year, in a contemporary Canadian cohort.

## Materials and methods

We performed an observational, retrospective cohort study that analyzed patient level data obtained by linking several provincial registries from Manitoba, Canada housed at the Manitoba Centre for Health Policy (MCHP) [10]. These databases included: the Manitoba Health Insurance Registry (patient registry and dates of coverage), Vital Statistics (date and cause of death), Medical Services (physician claims), Canadian Institute for Health Information (CIHI) Discharge Abstract Database (DAD) (hospital admission data), Diagnostic Services of Manitoba (DSM) (laboratory diagnostics), and the Drug Program Information Network (DPIN) database (drug prescriptions). Supplemental information on data availability is available in S1 File. We determined all primary and secondary diagnoses using ICD-9-CM and ICD-10-CM codes in both hospital discharge abstracts and medical services claims (S1 Table). All drugs were classified according to the Anatomical Therapeutic Chemical system (S2 Table). This study received ethics approval from the University of Manitoba Health Research Ethics Board (ethics file number HS18939).

### Study populations

#### Myocardial infarction (MI) population

All patients admitted to hospital with a diagnosis of MI (ICD-10 I21 [2]) between April 1^st^, 2006 and March 31^st^, 2010 were reviewed. Patient characteristics at baseline were established using hospitalization data, drug prescription claims, and medical services claims from January 1^st^, 2004 and onward. Patients who died within 7 days of the index MI were excluded from analyses. Follow-up data was collected from patients until November 30^th^, 2014 or death.

#### Early event free post-MI population

The early event free post-MI population included those from the original MI population who had no MI or stroke in the first 12 months after the index MI event, with a post-MI index date defined as the date of the index MI event plus 12 months.

#### High-risk subgroup

The high-risk subgroup was selected to mimic the characteristics of post MI patients evaluated in a recently completed clinical trial of DAPT. This subset included all individuals aged ≥ 50 with at least one of the following risk factors: age ≥ 65 at the 12-month post-MI index date, at least one MI prior to the index event, or a diagnosis of renal dysfunction prior to the 12-month post-MI index date, with the following exclusion criteria: history of stroke, dialysis 12 months prior to the index MI, or current use of oral anticoagulant therapy within 30 days of the index MI [8].

### Outcomes

The primary endpoint was major adverse cardiovascular events (MACE), defined as the composite of non-fatal MI (ICD10 I21), non-fatal stroke (ICD10 I61-I64), or CV death (cause of death attributed to ICD10-CM codes I00-I99). Secondary endpoints included the association of clinical factors as they relate to predicting CV outcomes and mortality. In addition, we ascertained overall hospitalization rates in the MI population.

### Statistical analyses

We performed descriptive analysis of included populations at the time of the index MI, at 365 days post MI in the early event free post-MI population, as well as in those included in the high-risk subgroup. We constructed Kaplan-Meier curves with the composite endpoint of a subsequent CV event (MI or stroke) or CV mortality during the first 365 days following the index MI event and from day 366 until the end of the study follow up, stratified by age in the entire population, the early event free post-MI population, and the high-risk subgroup and evaluated differences in survival between groups using the log-rank test.

In addition, we developed a Cox proportional hazards model to analyze the association between risk factors for CV disease (including age, sex, prior MI, prior stroke, diabetes, prior heart failure, prior unstable angina, and use of revascularization) and the composite endpoint. All analyses were performed using SAS Version 9.4.

## Results and discussion

### Patient populations and comorbid conditions

Between April 1^st^, 2006 and March 31^st^, 2010, 8,493 patients were admitted to a tertiary care centre in Manitoba with a diagnosis of acute MI and were alive at 7 days following admission. Of these patients, 6,749 (79.5%) survived without a subsequent MI or stroke for 365 days post-discharge, and 3,883 (57.5%) were part of the high risk subgroup. Median follow-up in all patients was 5.1 years (interquartile range 1.6–6.6).

Patients who survived the first year post MI were similar in age and sex to the entire MI cohort, and were less likely to have diabetes, heart failure or peripheral arterial disease at the time of their index event. They were more likely to undergo percutaneous coronary intervention (PCI), but had a similar rate of coronary artery bypass graft (CABG) surgery. The high-risk subgroup of the 1-year event free population was older and had more females than the comparison cohort. These high risk individuals had a lower rate of PCI, but a higher rate of CABG. Diabetes, hypertension and reduced kidney function were more common in this population (Table 1).

**Table 1:** Baseline demographic characteristics.

	All (n = 8493)	Early Event Free Post-MI Population (n = 6749)	High-Risk Subgroup (n = 3883)
Age (years)			
Mean (SD)	67.7 (14.0)	66.1 (13.8)	73.1 (10.4)
Median (IQR)	68.1 (56.9–79.1)	65.9 (55.5–77.1)	73.3 (66.1–81.0)
<50 years	985 (11.6%)	901 (13.4%)	0 (0%)
50–64 years	2684 (31.6%)	2343 (34.7%)	806 (20.8%)
65–74 years	1889 (22.2%)	1507 (22.3%)	1379 (35.5%)
75–84 years	1924 (22.7%)	1363 (20.2%)	1156 (29.8%)
≥85 years	1011 (11.9%)	635 (9.4%)	542 (14.0%)
Gender, n (%)			
Male	5581 (65.7%)	4519 (67.0%)	2398 (61.8%)
Female	2912 (34.3%)	2230 (33.0%)	1485 (38.2%)
Heart Failure, n (%)	1998 (23.5%)	1261 (18.7%)	901 (23.2%)
Previous MI, n (%)	193 (2.3%)	129 (1.9%)	102 (2.6%)
Previous Unstable Angina Pectoris, n (%)	370 (4.4%)	263 (3.9%)	208 (5.4%)
Peripheral Arterial Disease, n (%)	347 (4.1%)	216 (3.2%)	159 (4.1%)
PCI, n (%)	3285 (38.7%)	2987 (44.3%)	1410 (36.3%)
CABG, n (%)	776 (9.1%)	540 (8.0%)	460 (11.9%)
Stroke, n (%)	382 (4.5%)	255 (3.8%)	0 (0%)
Atrial Fibrillation, n (%)	1426 (16.8%)	973 (14.4%)	583 (15.0%)
Diagnosed Renal Dysfunction, n (%)	343 (4.0%)	202 (3.0%)	120 (3.1%)
Laboratory Renal Dysfunction, n (%)	1904 (22.4%)	1308 (19.4%)	968 (24.9%)
Diabetes, n (%)	2765 (32.6%)	1993 (29.5%)	1556 (40.1%)
Hypertension, n (%)	4822 (56.8%)	3723 (55.2%)	2468 (63.6%)
Moderate and Severe Liver Disease, n (%)	67 (0.8%)	49 (0.7%)	31 (0.8%)
Major Bleeding, n (%)	277 (3.3%)	201 (3.0%)	136 (3.5%)
Bleeding Diathesis/Coagulation Disease, n (%)	208 (2.5%)	142 (2.1%)	84 (2.2%)
Cancer, n (%)	1060 (12.5%)	772 (11.4%)	554 (14.3%)
Ongoing Medications (365 Days After Discharge):			
Statins	6316 (74.4%)	5211 (77.2%)	3038 (78.2%)
Beta Blockers	6212 (73.1%)	5043 (74.7%)	3002 (77.3%)
Angiotensin-Converting Enzyme-Inhibitors (ACEIs)	4916 (57.9%)	4053 (60.0%)	2318 (59.7%)
Angiotensin Receptor II Blockers (ARBs)	1353 (15.9%)	1085 (16.1%)	743 (19.1%)
Anti-diabetics	2101 (24.7%)	1561 (23.1%)	1196 (30.8%)
Anti-coagulants	776 (9.1%)	550 (8.2%)	222 (5.7%)
Anti-platelets (excluding ASA)	5725 (67.4%)	4685 (69.4%)	2673 (68.8%)

Invasive treatment rates differed between the entire MI population and the 1-year event free post-MI population, with 9.1% of the total MI population having received CABG for the index event vs. 8.0% in the early event free post-MI population. PCI was more common in the early event free post-MI population with a rate of 44.3% at the index event compared with 38.7% in the entire MI population. Statin use at baseline was marginally higher in those in the early event free post-MI population in comparison to the entire MI population (77.2% vs. 74.4%), anti-diabetic medication use was lower (23.1% vs. 24.7%), and anticoagulant use was lower (8.2% vs. 9.1%).

### Events

MACE occurred in 1,744 of the 8,493 patients in the MI population in the first 365 days following the index MI. During the first thirty days, 133 (1.6%) of the 8,493 patients died; 449 (5.3%) died during the first 150 days, and 827 (9.7%) died during the first year. Specifically, 484 of the 827 (58.5%) who died in the first year had a cause of death attributable to CV disease. Additionally, 1,058 (12.5%) of these patients had at least one recurrent MI, and 188 (2.2%) suffered a stroke.

**Fig 1:**
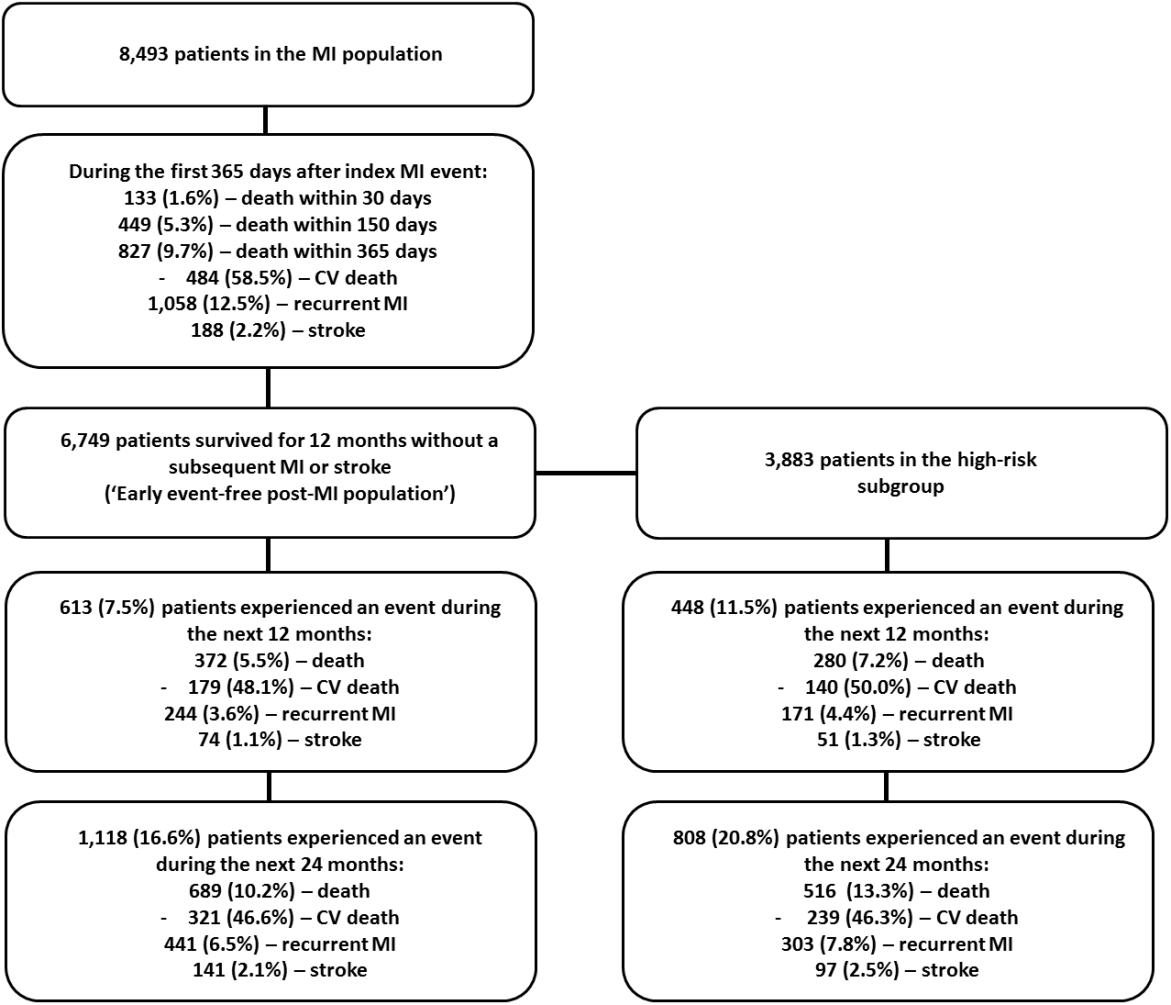
Overview of MI population outcomes.

Mortality declined, but was still substantial, in the following two years, with 372 (5.5%) of the remaining 6,749 patients in the early event free post-MI population dying in the next twelve months (48.1% CV deaths), 244 (3.6%) having a recurrent MI, and 74 (1.1%) suffering a stroke. Similar rates were observed in the third year. An overview of patient outcomes is shown in (Fig 1).

### Survival analyses

**Fig 2:**
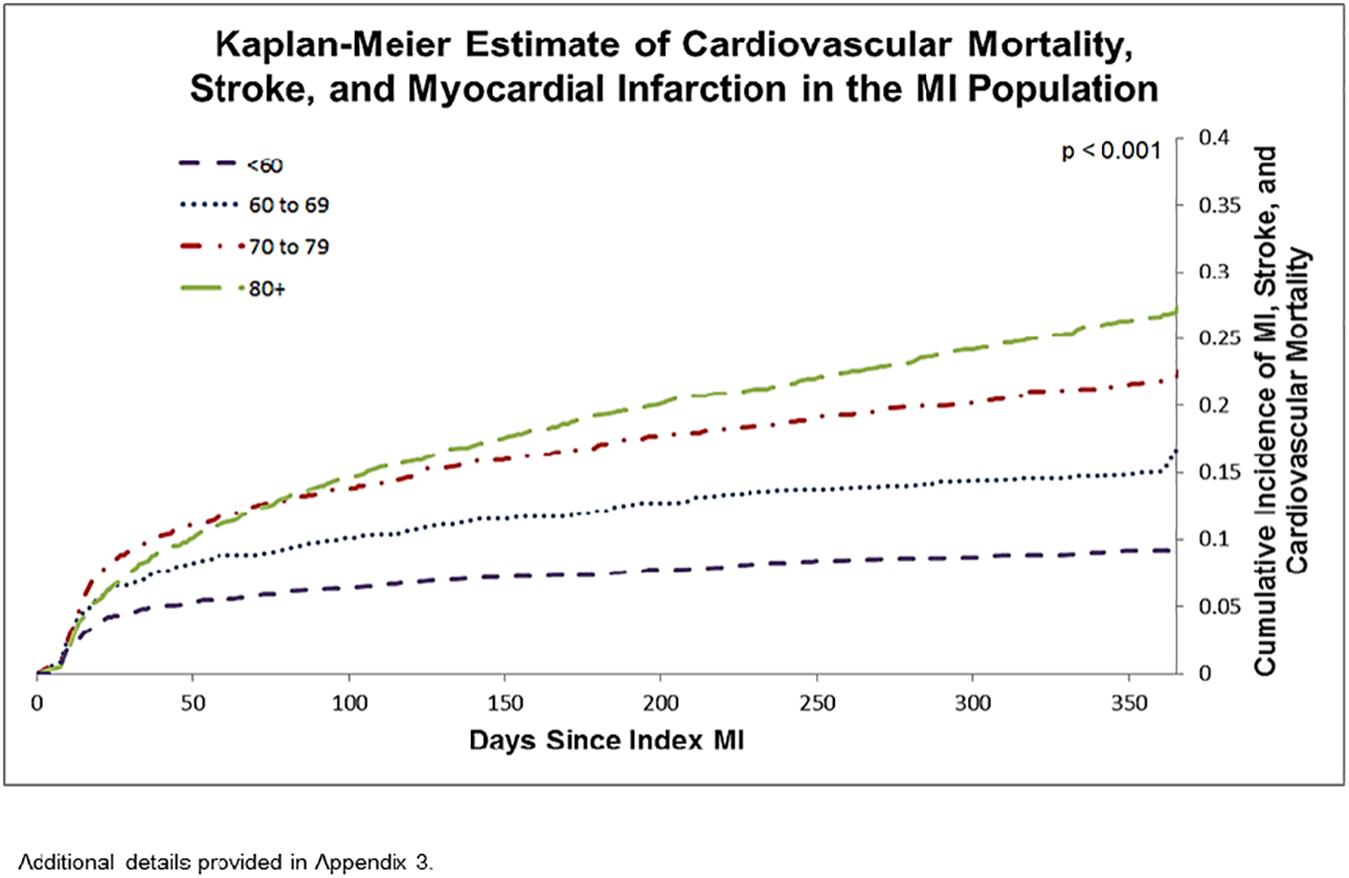
Kaplan-Meier estimate of cardiovascular mortality, stroke, and myocardial infarction in the MI population.

**Fig 3:**
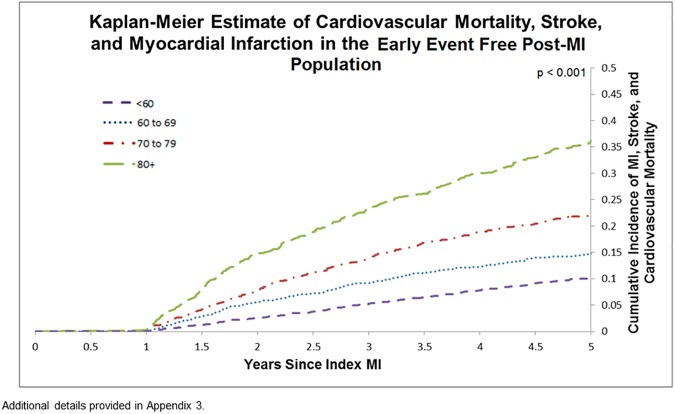
Kaplan-Meier estimate of cardiovascular mortality, stroke, and myocardial infarction in the early event free post-MI population.

The cumulative probability of the composite endpoint (subsequent MI, stroke, or CV mortality) increased with age in the entire cohort, with 9.3% of those less than 60 experiencing one of the above events in the first 365 days following the index event, to 27.0% in those aged 80+ (Fig 2) (p < 0.001). A similar trend was observed in the early event free post-MI population over the following four years, with 5.3% of those less than 60 years of age experiencing an event at 3 years, and 10.2% at 5 years, compared with 23.3% of those aged 80+ experiencing an event at 3 years, and 36.0% at 5 years (Fig 3) (p < 0.001).

**Fig 4:**
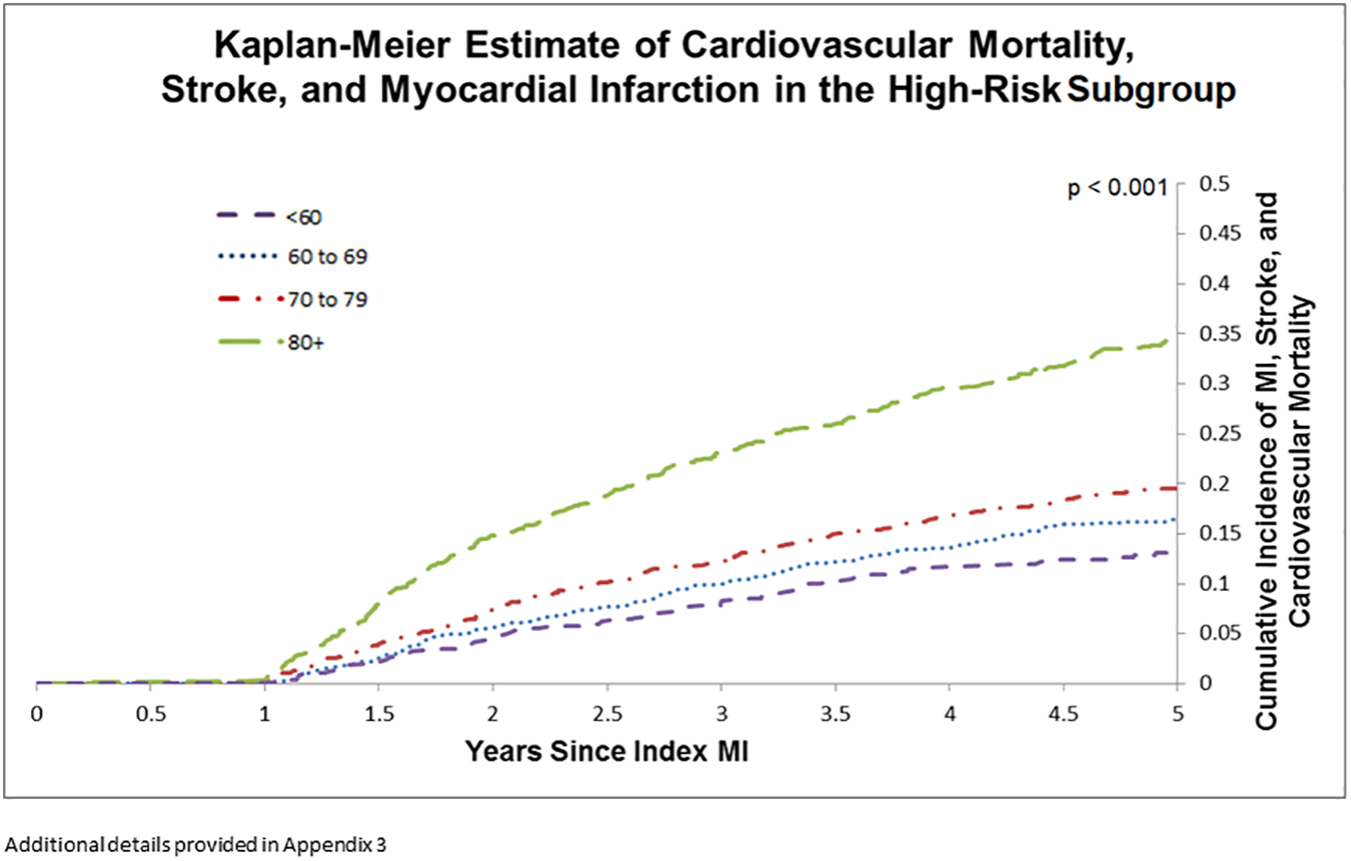
Kaplan-Meier estimate of cardiovascular mortality, stroke, and myocardial infarction in the high-risk subgroup.

While the cumulative incidence of the composite endpoint still increased with age in the high-risk subgroup, the difference was less pronounced among younger individuals. Three year incidence of CV mortality, MI, and stroke was 8.1% among those aged less than 60, 9.9% in those 60 to 69, 12.1% in those 70 to 79, and 23.0% among those aged 80+ (p < 0.001). 5-year incidence of MACE was 13.1% in those aged less than 60, 16.5% in those 60 to 69, 19.6% in those 70 to 79, and 34.4% among those aged 80+ (Fig 4) (p < 0.001). Additional information for survival analyses are provided in (S3 Table).

### Factors associated with MACE during follow-up

We found that older age, diabetes, prior MI, prior heart failure, and absence of revascularization were independently associated with an increase in MACE in the first 365 days following the index event. Between day 365 and the end of the study period, an independent increase in the composite endpoint was found for older age, male sex, diabetes, prior stroke (to the index MI date), prior heart failure, prior unstable angina, and absence of revascularization. Older age (>80), revascularization, and prior unstable angina were more strongly associated with longer term events, whereas prior MI was more strongly associated with MACE during the first year. These hazard ratios are presented in Table 2.

**Table 2:** Cox multivariable proportional hazards regression model of risk factors for the combined endpoint of cardiovascular death, subsequent MI, or stroke.

	First 365 Days After Index MI		Day 366 Until End of Study	
Variable	HR (95% CI)	*P-value*	HR (95% CI)	*P-Value*
Age 60–69 vs. Age < 60	1.47 (1.24–1.74)	*<* .*0001*	1.40 (1.19–1.65)	*<* .*0001*
Age 70–79 vs. Age < 60	1.98 (1.69–2.33)	*<* .*0001*	1.91 (1.63–2.23)	*<* .*0001*
Age ≥ 80 vs. Age < 60	2.29 (1.94–2.70)	*<* .*0001*	3.13 (2.68–3.66)	*<* .*0001*
Females vs. Males	0.91 (0.82–1.01)	*0*.*0834*	0.87 (0.78–0.97)	*0*.*0111*
Prior MI	1.51 (1.14–2.00)	*0*.*0038*	1.16 (0.82–1.64)	*0*.*4023*
Prior Stroke	1.19 (0.97–1.46)	*0*.*1036*	1.27 (1.02–1.57)	*0*.*0313*
Diabetes	1.58 (1.42–1.76)	*<* .*0001*	1.59 (1.42–1.77)	*<* .*0001*
Prior Heart Failure	1.90 (1.70–2.12)	*<* .*0001*	2.27 (2.03–2.54)	*<* .*0001*
Prior Unstable Angina	0.91 (0.73–1.14)	*0*.*4175*	1.27 (1.03–1.58)	*0*.*0287*
No Revascularization vs. Revascularization	1.39 (1.23–1.56)	*<* .*0001*	1.96 (1.75–2.20)	*<* .*0001*

### Hospitalizations

There was a total 36,848 total hospitalizations in the MI cohort over 37,223 total follow-up years, resulting in 0.99 hospitalizations per year of follow-up. The median number of hospitalizations following the index event was 3 per patient (interquartile range 2 to 6). Of these 36,848 hospitalizations, 70.8% (26,091) had at least one diagnosis that was associated with CV disease. Median hospital length of stay was 5 days (interquartile range 2–9). In total, following index event, the 8,493 MI patients experienced a total of 318,465 hospital days (37.5 days per patient), or 8.6 days of hospitalization per patient-year of follow-up.

## Discussion

In this provincial cohort study of patients who survived their index MI, nearly one in four patients reached the clinical endpoint of stroke, recurrent MI, or death in the first 365 days after the index event. Older age, diabetes, prior heart failure and absence of revascularization were associated with a poor prognosis in the first year post discharge. For patients who survived for 12 months without a subsequent MI event or stroke (the early event free post-MI population), one in ten experienced an event within the following year with a mortality rate exceeding 5%. Although this early event free post-MI population was at lower risk of an event when compared to the entire MI population, the mortality risk is still substantial and higher than risk thresholds for DAPT proposed by the American Heart Association and European Society of Cardiology guidelines [11, 12].

The findings of this study are consistent with those of other retrospective analyses in Western countries looking at outcomes following acute MI. A previously published study from Europe found an 18.3% risk of the composite of CV death, recurrent MI, or stroke during the first-year post-MI which is comparable to the 17.5% observed in our population. Additionally, in the following 3 years during this stable period, they noted a 20.0% cumulative incidence of MACE, comparable to a 15.3% rate in our population. Our population was substantially younger (71.5 years versus 66.1 years), and this may have contributed to the lower cumulative incidence during follow-up. Older age, male sex, diabetes, histories of stroke and heart failure were consistently associated with a higher hazard of MACE in our study and in the European cohort [2, 13, 14].

Our findings may have implications regarding recommendations for the optimal length of prescribing DAPT following MI. A previous meta-analysis of randomized controlled trials of long-term DAPT found that, over a mean follow-up of 31 months, there remains a significant benefit from the continuation of DAPT, with a reduction in the occurrence of several adverse CV events (e.g., stroke, thrombosis, recurrent MI). The authors found no change in all-cause mortality with extended DAPT, but did find an increase in the overall risk of major bleeding [7, 15]. Consideration of this therapy in post-MI patients must therefore consider the tradeoff between risks of increased bleeds versus the potential benefits to adverse CV events.

In order to facilitate these treatment decisions, risk scoring algorithms such as the “DAPT score” could be used to triage patients into appropriate treatment regimens and have been recommended as Class II evidence by the American College of Cardiology [9]. Extension of DAPT prescription in highest risk patients using the “DAPT score” successfully reduced the risk of ischemic events at the cost of a small, statistically insignificant increase in bleeding [16].

This study may also help inform the economic outcomes following acute MI. A costing study has shown that health care costs increase almost two-fold per year following a MI among early event free (1 year) adults aged greater than 65 in the United States, and remain consistently elevated for at least five years [17]. Choosing the appropriate patients in which to extend therapy could be further clarified with the development of decision analytical models considering risk thresholds and the potential adverse outcomes of long term DAPT in a Canadian context.

It is also important to note that less than 50% of deaths in our follow-up period were from CV causes. These findings suggest that prevention strategies which solely focus on prevention of stroke or MI are inadequate by themselves for a meaningful extension of life. Alternative strategies, such as exercise programs, in Canadian post-MI patients have been shown to have a positive benefit on CV and all-cause mortality [18]. Strategies with a multidisciplinary approach focusing on non-CV related comorbid conditions common in this patient population, such as diabetes, cancer, and renal and liver disease, may be critical to improve overall survival, and warrant further consideration [14].

This study had several strengths. Primarily, we were able to provide a representative sample of the Manitoba population using administrative databases that captured all hospitalizations for MI in the region, and the majority of medical services claims. In addition, we were able to capture all medication prescriptions via the Drug Program Information Network which maintains a database of all behind-the-counter dispersions in Manitoba.

There were also several limitations to our analyses. First, there is potential that some primary care encounters would have been missed by the available administrative claims databases, as some primary care providers are paid through alternative funding arrangements in Manitoba [19], particularly for physicians in rural communities, which are also areas of Manitoba that have a disproportionately high burden of chronic disease [20]. Second, we were unable to accurately measure acetylsalicylic acid/aspirin use because many patients obtain this medication over the counter, and thus this information would not be captured by the Drug Program Information Network (DPIN) database. Third, informative covariates such as smoking status and body mass index were not available in our data. Lastly, we only had data leading back to January 1^st^, 2004, and as such, the true rates of prior events (such as MI, heart failure, unstable angina, and stroke) are likely underestimated in our analysis.

## Conclusions

The risk of adverse events remains consistently elevated among MI patients in the years following their index MI event. Interventions to more intensively monitor, evaluate, and treat these patients should be considered beyond the first year following myocardial infarction.

## Supporting information

S1 Table ICD9 and ICD10 codes used to classify comorbid conditions. *NOTE: Diabetes classification also includes those who received a prescription for an anti-diabetic (ATC code A10), and hypertension includes those who received a prescription for an anti-hypertensive (e.g., ACEI or ARB) (ATC code C09)–See S2 Table. Criteria for laboratory diagnosed renal dysfunction included a lab test with an estimated glomerular filtration rate (eGFR) less than 60 ml/min/1.73m^2^ or a urine albumin-to-creatinine ratio greater than 3 mg/mmol.
(DOCX)

S2 Table Anatomical therapeutic chemical (ATC) codes used to classify medications. (DOCX)

S3 Table Kaplan–Meier estimates of cumulative incidence. (DOCX)

S1 File Data availability statement. (DOC)
