# Supplementary material for: Long term health outcomes in patients with a history of myocardial infarction: A population based cohort study
Source: PLoS One. 2017 Jul 12;12(7):e0180010. doi: 10.1371/journal.pone.0180010 (PMC5507480; doi:10.1371/journal.pone.0180010)
Supplement: S1 Table — *NOTE: Diabetes classification also includes those who received a prescription for an anti-diabetic (ATC code A10), and hypertension includes those who received a prescription for an anti-hypertensive (e.g., ACEI or ARB) (ATC code C09)–See S2 Table. Criteria for laboratory diagnosed renal dysfunction included a lab test with an estimated glomerular filtration rate (eGFR) less than 60 ml/min/1.73m2 or a urine albumin-to-creatinine ratio greater than 3 mg/mmol. (DOCX) [file pone.0180010.s001.docx]

**S1 Table. ICD9 and ICD10 codes used to classify comorbid conditions**

| **Variable** | **ICD9 Codes (medical services)** | **ICD10 Codes (hospitalizations)** |
| --- | --- | --- |
| **Heart Failure** | 428 | I110 / I130 / I132 / I50 |
| **Myocardial Infarction** | 410 | I21 |
| **Unstable Angina Pectoris** | 413 | I20 |
| **Peripheral Arterial Disease** | 443 | I70 / I73 / I74 |
| **Stroke** | 430 / 431 / 433 / 434 / 435 / 436 | I61 – I64 |
| **Atrial Fibrillation** | 427 | I48 |
| **Diagnosis of Renal Dysfunction** | 585 / 753 | I150 / I151/ N03 – N05 / N11 / N184 / N185 / Q60 / Q61 / Z491 / Z992 |
| ***Diabetes** | 250 | E10 – E14 / Z794 |
| ***Hypertension** | 401 | I10 |
| **Moderate and Severe Liver Disease** | 571 | K71 / K721 / K73 – K76 / R18 |
| **Major Bleeding** | 431 | D629 / I60 – I62 / I650 / K250 / K252 / K254 / K260 / K262 / K264 / K265 K266 / K270 / K272 / K274 / K276 / K280 / K282 / K284 / K286 / K290 / K291 / K292 |
| **Bleeding Diathesis/Coagulation Disease** | 286 | D66 – D67 / D680 – D684 / D688 / D689 / D691 / D693 –D696 |
| **Cancer** | 140-239 | All C codes |

*NOTE: Diabetes classification also includes those who received a prescription for an anti-diabetic (ATC code A10), and hypertension includes those who received a prescription for an anti-hypertensive (e.g., ACEI or ARB) (ATC code C09) – See S2 Table.

**Intervention codes used to classify PCI (percutaneous coronary intervention) and CABG (coronary artery bypass graft)**

| **Intervention** | **Intervention Code** |
| --- | --- |
| **PCI** | 1IJ50 / 1IJ57 |
| **CABG** | 1IJ76 |

Criteria for laboratory diagnosed renal dysfunction included a lab test with an estimated glomerular filtration rate (eGFR) less than 60 ml/min/1.73m^2^ or a urine albumin-to-creatinine ratio greater than 3 mg/mmol.
