# Supplementary material for: Long term health outcomes in patients with a history of myocardial infarction: A population based cohort study
Source: PLoS One. 2017 Jul 12;12(7):e0180010. doi: 10.1371/journal.pone.0180010 (PMC5507480; doi:10.1371/journal.pone.0180010)
Supplement: S2 Table — (DOCX) [file pone.0180010.s002.docx]

**S2 Table. Anatomical therapeutic chemical (ATC) codes used to classify medications**

| **Medication Type** | **ATC Codes** |
| --- | --- |
| Statins | C10AA |
| Beta Blockers | C07 |
| Angiotensin-Converting Enzyme-Inhibitors (ACEIs) | C09A / C09B |
| Angiotensin Receptor II Blockers (ARBs) | C09C / C09D |
| Anti-diabetics | A10 |
| Anti-coagulants | B01AA / B01AB / B01AD / B01AE / B01AF / B01AX |
| Anti-platelets | B01AC |
