# Supplementary material for: Long term health outcomes in patients with a history of myocardial infarction: A population based cohort study
Source: PLoS One. 2017 Jul 12;12(7):e0180010. doi: 10.1371/journal.pone.0180010 (PMC5507480; doi:10.1371/journal.pone.0180010)
Supplement: S3 Table — (DOCX) [file pone.0180010.s003.docx]

**S3 Table. Kaplan – Meier estimates of cumulative incidence**

1. **In the entire MI population by age**

| **Age** | **100 days** | **180 days** | **365 days** |
| --- | --- | --- | --- |
| **<60** | 0.0638 | 0.0736 | 0.0925 |
| **60 to 69** | 0.1011 | 0.1212 | 0.1512 |
| **70 to 79** | 0.1373 | 0.1701 | 0.2202 |
| **80 +** | 0.1459 | 0.1932 | 0.2696 |
| **Overall** | 0.1079 | 0.1338 | 0.1750 |

1. **In the early event free post-MI population by age**

| **Age** | **2 years** | **3 years** | **4 years** | **5 years** |
| --- | --- | --- | --- | --- |
| **<60** | 0.0258 | 0.0527 | 0.0778 | 0.1017 |
| **60 to 69** | 0.0557 | 0.0918 | 0.1224 | 0.1471 |
| **70 to 79** | 0.0784 | 0.1384 | 0.1892 | 0.2205 |
| **80 +** | 0.1466 | 0.2331 | 0.3006 | 0.3601 |
| **Overall** | 0.0672 | 0.1142 | 0.1533 | 0.1846 |

1. **In the high-risk subgroup by age**

| **Age** | **2 years** | **3 years** | **4 years** | **5 years** |
| --- | --- | --- | --- | --- |
| **<60** | 0.0453 | 0.0809 | 0.1173 | 0.1307 |
| **60 to 69** | 0.0562 | 0.0990 | 0.1351 | 0.1645 |
| **70 to 79** | 0.0732 | 0.1211 | 0.1683 | 0.1964 |
| **80 +** | 0.1476 | 0.2301 | 0.2962 | 0.3443 |
| **Overall** | 0.0861 | 0.1405 | 0.1881 | 0.2194 |
