# Supplementary material for: Long term health outcomes in patients with a history of myocardial infarction: A population based cohort study
Source: PLoS One. 2017 Jul 12;12(7):e0180010. doi: 10.1371/journal.pone.0180010 (PMC5507480; doi:10.1371/journal.pone.0180010)
Supplement: S1 File — (DOC) [file pone.0180010.s004.doc]

**S1 File - Data availability statement**

Data are available from the Manitoba Centre for Health Policy, University of Manitoba for researchers who meet the criteria for access to confidential data. Data included is housed at Manitoba Health, Vital Statistics Manitoba, Diagnostic Services of Manitoba, and the Winnipeg Regional Health Authority.

All data used in our analysis is publicly available and is held by the Government of Manitoba or contracted agencies (e.g. laboratory services). Access to this data requires approval by the University of Manitoba Health Research Ethics Board and the Government of Manitoba’s Health Privacy Information Committee. Legal restrictions prevent the use of this data without approval from each of the individual trustees.

All of this data is provided unidentifiable and linked through a remote access server maintained by the Manitoba Centre for Health Policy (<http://umanitoba.ca/faculties/health_sciences/medicine/units/chs/departmental_units/mchp/>).
Email address: [info@cpe.umanitoba.ca](mailto:info@cpe.umanitoba.ca)

Specific data sets used in this project include:

- 1. Diagnostic Services of Manitoba (lab tests)

Diagnostic Services Manitoba, 1502-155 Carlton Street, Winnipeg, Manitoba, R3C 3H8, 1-204-926-8005

Information available at:

<http://umanitoba.ca/faculties/health_sciences/medicine/units/chs/departmental_units/mchp/resources/repository/descriptions.html?ds=DSM>

- 1. Manitoba Renal Program (dialysis status)

Health Information Privacy Committee, Manitoba Health, Seniors and Active Living , 4044-300 Carlton Street, Winnipeg, Manitoba, R3B 3M9 and the Section of Nephrology, University of Manitoba

Information available at:

<http://www.kidneyhealth.ca/wp/>

- 1. Hospital Discharge Abstracts (hospitalizations)

Health Information Privacy Committee, Manitoba Health, Seniors and Active Living , 4044-300 Carlton Street, Winnipeg, Manitoba, R3B 3M9

Information available at:

<http://umanitoba.ca/faculties/health_sciences/medicine/units/chs/departmental_units/mchp/resources/repository/descriptions.html?ds=Hospital>

- 1. Manitoba Health Insurance Registry (registration coverage periods, demographics)

Health Information Privacy Committee, Manitoba Health, Seniors and Active Living, 4044-300 Carlton Street, Winnipeg, Manitoba, R3B 3M9

Information available at:

<http://umanitoba.ca/faculties/health_sciences/medicine/units/chs/departmental_units/mchp/resources/repository/descriptions.html?ds=Insurance>

- 1. Manitoba Health Medical Services (physician claims/billings)

Health Information Privacy Committee, Manitoba Health, Seniors and Active Living, 4044-300 Carlton Street, Winnipeg, Manitoba, R3B 3M9

Information available at:

<http://umanitoba.ca/faculties/health_sciences/medicine/units/chs/departmental_units/mchp/resources/repository/descriptions.html?ds=MedicalClaims>

- 1. Drug Program Information Network – DPIN (pharmacy dispensing)

Health Information Privacy Committee, Manitoba Health, Seniors and Active Living , 4044-300 Carlton Street, Winnipeg, Manitoba, R3B 3M9 and the Section of Nephrology, University of Manitoba

Information available at: <http://umanitoba.ca/faculties/health_sciences/medicine/units/chs/departmental_units/mchp/resources/repository/descriptions.html?ds=DPIN>

- 1. Vital Statistics (mortality and cause of death)

Vital Statistics Agency, 254 Portage Avenue, Winnipeg, Manitoba, R3C 0B6, 1-204-945-3701

Information available at:

<http://umanitoba.ca/faculties/health_sciences/medicine/units/chs/departmental_units/mchp/resources/repository/descriptions.html?ds=Vital>
